# Position statement on longitudinal cracks and fractures of teeth

**DOI:** 10.1111/iej.14186

**Published:** 2025-01-22

**Authors:** Shanon Patel, Peng‐Hui Teng, Wan‐Chuen Liao, Matthew Craig Davis, Ales Fidler, Franziska Haupt, Cristiano Fabiani, Ronald Ordinola Zapata, Rahul Bose

**Affiliations:** ^1^ Guy's & St Thomas NHS Foundation Trust London UK; ^2^ Centre for Oral, Clinical & Translational Sciences King's College London London UK; ^3^ Specialist Practice London UK; ^4^ Unit of Endodontology & Endodontics, Faculty of Dentistry Universiti Kebangsaan Malaysia Kuala Lumpur Malaysia; ^5^ School of Dentistry, College of Medicine, National Taiwan University Taipei Taiwan; ^6^ Private Practice in Endodontics Winnetka Illinois USA; ^7^ Department of Endodontics and Restorative Dentistry, Faculty of Medicine University of Ljubljana Ljubljana Slovenia; ^8^ Department of Restorative Dentistry and Endodontics University Medical Centre Ljubljana Ljubljana Slovenia; ^9^ Department of Preventive Dentistry, Periodontology and Cariology University Medical Center Göttingen Göttingen Germany; ^10^ Specialist Practice Rome Italy; ^11^ Department of Endodontics University of Minnesota Minneapolis Minnesota USA

**Keywords:** cracked tooth, longitudinal fractures, split tooth, vertical root fracture

## Abstract

This position statement is a consensus view of an expert committee convened by the European Society of Endodontology (ESE). The statement is based on current clinical and scientific evidence as well as the collective reflective practice of the committee. The aim is to provide clinicians with evidence‐based, authoritative information on the aetiology, clinical presentation, and management of cracks and fractures that typically manifest along the long axis of the crown and/or root.

## INTRODUCTION

**FIGURE 1:**
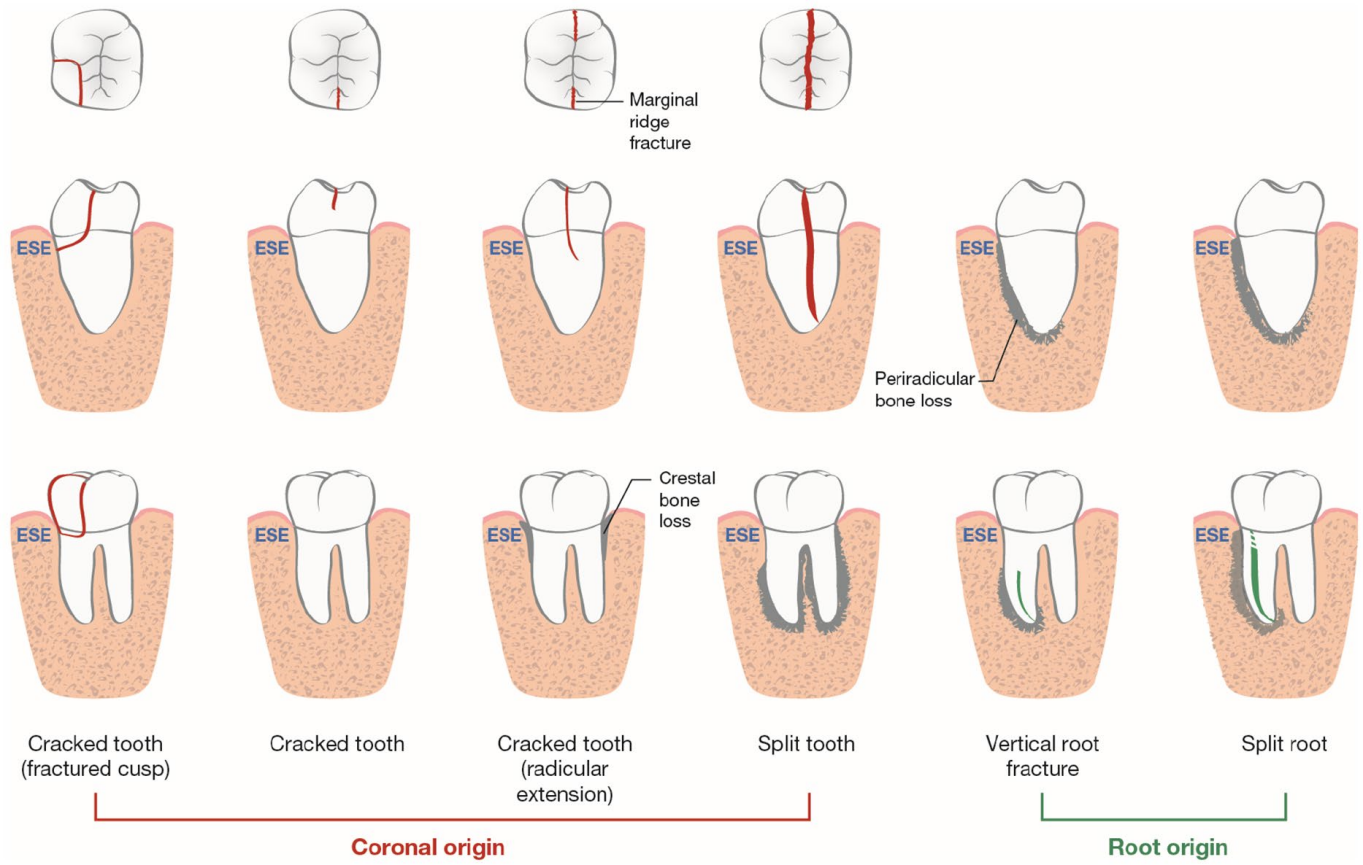
Common presentation of cracked tooth, split tooth, vertical root fracture, and split root. Top row: occlusal, middle row: mesio‐distal, lower row: bucco‐lingual aspects of a mandibular molar.

The incidence of cracked teeth (CT) and vertical root fractures (VRF) is increasing (American Association of Endodontists, 2022; Pan et al., 2022). The term ‘Longitudinal’ encompasses cracks and fractures that are vertical in nature and may progress over time (Figure 1 and Table 1) (Rivera & Walton, 2015). If left untreated, CT may progress to irreversible pulpal disease and possibly tooth loss. A VRF commonly progresses subtly, leading to a delayed diagnosis that results in extraction.

**TABLE 1:** Definitions of longitudinal cracks and fractures of teeth.

Craze Line	Superficial, coronal crack line confined to the enamel with no compromise of the integrity of the tooth.
Cracked Tooth (CT)	A crack extending into the dentine, of unknown depth or size, may extend subcrestally into the root. There may be pulpal involvement.
Fractured (cracked) Cusp	*Molars*‐(in) complete fracture extending down a bucco/lingual groove and mesio‐distal direction of the affected cusp. *Premolars*‐usually involve marginal ridges. +/− pulpal involvement.
Split Tooth (ST)	Complete, visible separation of the entire tooth into 2 parts, usually in a mesio‐distal direction
Vertical root fracture (VRF)	An incomplete longitudinal (axial) root fracture, involving the cementum, dentine, and root canal space.
Split Root	A complete VRF with visible separation of the root into distinct segments.

Timely identification and appropriate management are essential to increase the life span of the affected tooth.

## CRACKED TEETH

A crack extending into the dentine, of unknown depth or size, which may extend subcrestally into the root. There may be pulpal involvement.

### Prevalence

The prevalence of coronal (visible) CT has been reported to be as high as 70% (Hilton et al., 2011). Posterior teeth (maxillary (pre)molars and mandibular molars) are more commonly affected by cracks (Hilton et al., 2017; Krell & Caplan, 2018; Yang et al., 2017). Historically, cracks were associated with restored teeth (Cameron, 1976; Kahler, 2008); however, over the last two decades studies have reported an increasing incidence of cracks in unrestored teeth (Alaugaily & Azim, 2022; Kang et al., 2016; Lee et al., 2021).

CT incidence increases with age and is most prevalent in patients over 40 years of age (Hilton et al., 2017). Age‐related changes in the biomechanical properties of dentine result in an increasing mineral‐to‐collagen and cross‐linking ratio that renders the radicular dentine less resilient and more susceptible to crack propagation (Chen et al., 2023; Montoya et al., 2015). A significant increase in the prevalence of CT has been reported since the Coronavirus disease (COVID‐19) pandemic (Mirhashemi et al., 2022; Nocini et al., 2023; Nosrat et al., 2022).

### Aetiology

CT aetiology is multifactorial, involving occlusal interferences, tooth morphology, previous operative dentistry, increased masticatory loading due to hard food diets, and/or parafunctional habits (Table 2).

**TABLE 2:** Potential predisposing and contributory factors for cracked teeth and vertical root fractures (VRF).

	Cracked teeth	VRF in non‐root‐filled teeth	VRF in root‐filled teeth
Clinical features	*Predisposing factors* Compromised structural integrity Changes in dentinal biomechanical properties due to ageing Specific cuspal anatomy (functional cusp, buccal‐lingual dimension, cuspal inclination, etc.) Posterior location of the tooth Parafunction and/or unfavourable occlusal arrangement. Dietary habit *Contributory factors* Stress generated from restorative procedures Thermal expansion, contraction, and/or corrosion of restorative materials	*Predisposing factors* Presence of pre‐existing (micro) cracks and fractures Changes in dentinal biomechanical properties due to ageing Specific root canal anatomy and morphology (narrow mesiodistal width, ovoid shape canal) Posterior location of the tooth Parafunction and/or unfavourable occlusal arrangement. Dietary habit	*Predisposing factors* Compromised structural integrity Pre‐existing (micro)cracks and fractures Changes in dentinal biomechanical properties due to ageing and/or endodontic treatment Specific root canal anatomy and morphology (e.g. narrow mesiodistal width, ovoid shape canal) Specific cuspal anatomy (functional cusp, buccal‐lingual dimension, cuspal inclination etc.) Posterior location of the tooth Parafunction and/or unfavourable occlusal arrangement. *Contributory factors* Excessive removal of sound dentine during RCT Prolonged exposure to intracanal medicaments and disinfectants. Inappropriate execution of post‐endodontic restoration (including post preparation) Excessive pressure generated during root canal obturation Root end preparation with ultrasonic retro‐tips

The apical propagation of a dentinal crack occurs over a period of prolonged cyclical loading, which may result in the ingress of microbes into the pulp chamber, culminating in pulp necrosis (Ricucci et al., 2015).

### Clinical features

The clinical presentation varies depending on the location, size, and extension of the crack(s), as well as the pulpal, periapical, and periodontal involvement (Table 3).

**TABLE 3:** Clinical and radiographic features.

	Craze line	Fractured cusp	Cracked teeth	VRF in non‐root filled teeth	VRF in root filled teeth	Split tooth
Clinical features	Typically asymptomatic Craze line blocking transilluminated light	*Early stage* Thermal sensitivity Short sharp pain upon mastication or releasing from pressure *Advanced stage* Symptoms may be relieved when the cusp fractures off Separated cuspal fragment May develop pulpitis or apical periodontitis symptoms if fracture involves pulp	*Early stage* Attrited occlusal surface +/− symptoms (e.g., thermal sensitivity, pain upon mastication or releasing from pressure) Positive response to the sensibility test Direct visualization of craze/crack line(s) blocking transilluminated light *Advanced stage* May elicit negative response to the sensibility test +/− symptoms of pulpitis or apical periodontitis (e.g., dull throbbing pain, tenderness to percussion and/or palpation, abscess etc.) Usually either buccal or lingual cups will be tender to pecussion +/− isolated, deep, narrow periodontal pocket	*Early stage* Attrited occlusal surface +/− symptoms (e.g., thermal sensitivity, pain or discomfort upon mastication, or persistent ache) Positive response to the sensibility test *Advanced stage* Negative response to the sensibility test +/− symptoms of apical periodontitis (e.g., dull throbbing pain, tenderness to percussion and/or palpation, abscess etc.) Direct visualization of a fracture Mimicking periodontal disease +/− isolated, deep, narrow periodontal pocket Mobility	+/− symptoms of apical periodontitis (e.g., pain upon mastication, tenderness to percussion and/or palpation, abscess etc.) Mimicking periodontal disease +/− isolated, deep, narrow periodontal pocket Direct visualization of a fracture Presence of a sinus/multiple sinuses close to gingival margin Mobility	Pain upon mastication Periodontal attachment loss and/or abscess Mobility
Radiographic features	Unremarkable	Unremarkable	*Early stage* Not remarkable May have subtle radiolucent lines in the coronal tooth structure *Advanced stage* +/− periapical radiolucency in necrotic teeth	*Early stage* No obvious changes +/− subtle crestal bone loss Radiographic density changes or unusual radiolucent lines in the root canal Abnormal widening of the root canal space Subtle periodontal ligament widening or periradicular bone loss on the lateral root walls *Advanced stage* Separated root fragments Periradicular bone loss adjacent to the VRF (Halo or J‐shaped radiolucency)	*Early stage* No obvious changes +/− subtle crestal bone loss Radiographic density changes or unusual radiolucent lines in the root filling Subtle periodontal ligament widening or periradicular bone loss on the lateral root walls *Advanced stage* Separated root fragments Periradicular bone loss adjacent to the VRF (Halo or J‐shaped radiolucency)	Separated tooth fragments Periradicular bone loss

A practice‐based study of 2858 teeth from 209 dentists in the USA reported that 45% of CT were symptomatic, the most common symptoms being pain to cold (37%), biting pain (16%), and spontaneous pain (11%) (Hilton et al., 2018). Tenderness to percussion and marginal fracture lines are also commonly reported findings (de Toubes et al., 2022).

Diagnosis of early‐stage CT can be challenging due to poorly localised symptoms, which may be misdiagnosed as (non‐)odontogenic conditions, or in some cases CT may be an asymptomatic, incidental finding (Hilton et al., 2020). At a later stage, a discernible crack line on interproximal marginal ridges or buccal/lingual aspects may be seen. The patient may also develop a fractured cusp as a result of a crack propagation.

Clinical examination of the suspected CT should include appropriate sensibility tests, tooth mobility, and assessment of the surrounding soft tissues. Occlusal assessment may reveal indirect signs of increased (para)functional forces; these include wear facets, loss of canine guidance, and/or occlusal interferences (Table 3). Narrow periodontal probing of less than 6 mm is associated with CT, whereas periodontal probing depths of VRF are deeper (Alaugaily & Azim, 2022).

In vital teeth, detection of a crack is aided by a bite test (eg., tooth sleuth) (Hilton et al., 2020). The aim of the bite test is to reproduce the patient's symptoms, particularly sudden sensitivity and sharp pain when biting hard foods and/or clenching, which ceases upon the release of pressure. In non‐vital teeth, there may be symptoms and/or signs of apical periodontitis (AP) as well as evidence of marginal ridge fractures.

Removal of existing restorations will facilitate visualisation of the nature of the crack (Abbott, 2004). Fibre optic transillumination and/or the use of stains such as methylene blue are recommended. The use of a dental operating microscope or loupes is critical in detecting dentinal cracks, as well as distinguishing them from craze lines (Clark et al., 2003; Kim et al., 2020). CT should be differentiated from craze lines, which are confined to the enamel, naturally occurring through mastication and becoming more prominent with age (Rivera & Walton, 2015). Craze lines are readily visible on anterior teeth but also evident on marginal ridges and/or axial surfaces of posterior teeth. Treatment of craze lines is not indicated, except for aesthetic reasons, i.e., stained craze lines.

### Radiographic features

The radiographic appearance (Table 3) on periapical radiographs (PRs) can be highly variable. Early‐stage CT may include no obvious signs of a crack and/or pathology. Hilton et al. (2017) reported that only 2% of CTs with vital pulps had evidence of a crack on a radiograph.

Radicular extension of a crack may result in (subtle) crestal vertical bone loss associated with pulpal necrosis. With long‐standing CT, there may be evidence of periapical lesions, thickening of the periodontal ligament, and/or radiolucency along the lateral aspect of the root.

CBCT may be indicated if clinical and/or PR assessments are inconclusive (American Association of Endodontists/American Academy of Oral & Maxillofacial Radiology, 2015; European Society of Endodontology, 2014). CBCT is not predictable in detecting cracks but may reveal subtle crestal bone loss associated with CT (Alaugaily & Azim, 2022; Gao et al., 2020).

### Management options

#### Clinically normal pulp and normal apical tissue

Shallow cracks and/or low risk of crack propagation may be periodically reviewed (Chen et al., 2023). It remains unclear for how long asymptomatic, untreated CT can remain stable and without further crack propagation, as current studies have only monitored cracks for 1–3 years (Ferracane et al., 2022; Hilton et al., 2020). When indicated, patients should be given advice on managing parafunctional habits. Occlusal interferences should also be managed to create a more harmonious occlusal scheme to limit extension of CT; this may require a multi‐disciplinary approach.

Untreated CT in high‐risk teeth may progress to a fractured cusp or a split tooth (see below), for example, the distal marginal ridge of last‐standing (pre‐)molars, an extensively restored tooth, a lone/last‐standing tooth, and/or patients with chronic parafunctional habits. In these cases, active treatment is indicated, e.g., cuspal coverage restoration, to reduce the likelihood of the crack propagating, thus increasing the longevity of the tooth (see VRF section below).

#### Reversible pulpitis

CT associated with reversible pulpitis may be monitored or (in‐)completely traced out depending on extent and restored with a composite restoration or a cuspal coverage restoration (American Association of Endodontists, 2022; Hilton et al., 2017). Treatment (type) is dependent on several factors and should be determined on a case‐by‐case basis. In some instances, it will not be pragmatic to trace out the entire crack, as this may result in inadvertent removal of a significant amount of sound tooth tissue and/or render the tooth unrestorable.

The decision on the type (and extent) of the restoration must be tailored to each patient's unique characteristics, rather than taking a ‘one fits all’ approach. Factors that may indicate a cuspal coverage restoration include the extent of the crack, history of spontaneous pain, moderate/significant caries, existing direct restorations, pain on biting, volume of residual coronal tooth structure, proximal contacts, as well as indirect factors such as occlusal scheme and parafunctional habits should be considered (Ferracane et al., 2022; Kakka et al., 2022).

A single‐stage approach, i.e., immediate definitive restoration, or a multiple‐stage approach, i.e., interim treatment (temporary crowns, direct composite splint) and review of pulpal status prior to definitive restoration, may be selected (Banerji et al., 2014; Ehrmann & Tyas, 1990; Kakka et al., 2022). While interim treatment allows pulpal healing and confirms initial diagnosis prior to definitive restoration, pulp vitality may be compromised due to microleakage, cement breakdown, and/or further definitive treatment (Kang et al., 2016; Wu et al., 2019).

There is no clear evidence on the most suitable restorative treatment approach to manage CT (de Toubes et al., 2022; Lee et al., 2021). CT managed with direct bonded composite restorations may be more likely to require root canal treatment and/or further repair of fractured restorations compared with CT managed with cuspal coverage restorations (Signore et al., 2008; Wu et al., 2019; Zhang et al., 2024). The reported incidence of endodontic intervention after restorative management has been reported to be between 7.7% and 20% (Krell & Rivera, 2007; Opdam et al., 2008).

#### Irreversible pulpitis/pulp necrosis

The survival rate of root filled with irreversible pulpitis, pulp necrosis, or pulp exposure following root canal treatment ranges from 84% to 88% over 1–5 years of follow‐up, and the success rate ranges from 76% to 82% after 1–2 years (Chen et al., 2021; Leong et al., 2020; Olivieri et al., 2020).

A 5 + mm periodontal probing is associated with reduced survival rates (Kang et al., 2016; Olivieri et al., 2020). Other negative prognostic factors include the presence of multiple cracks, radicular extension of cracks, location in the arch (terminal tooth), preoperative presence of AP, and/or placement of an intracanal post (Chen et al., 2021; Krell & Caplan, 2018; Leong et al., 2020).

Historically, radicular cracks extending beyond the root canal entrance have been regarded as having an unfavourable prognosis (Malentacca et al., 2021; Sim et al., 2016). Placing an intracanal barrier apical to the crack after endodontic treatment has been completed could increase the fracture resistance and provide a good seal from further microbial penetration. Davis and Shariff (2019) reported that this approach in combination with a cuspal coverage restoration and careful management of the occlusion had a 4‐year survival rate of 96.6%.

### Conclusion and summary

Current evidence suggests encouraging outcomes for vital as well as endodontically treated CT restored with cuspal coverage restorations. Early management, cuspal coverage restorations, and absence of deep periodontal probing depth of non‐endodontic origin increase the survival rate of CT management. Careful occlusal assessment and management of parafunctional habits and/or occlusal interferences is essential to reduce the likelihood of the propagation of existing cracks, thus improving the longevity of CT. Due to the broad variability in presentation, the term ‘CT syndrome’ should be avoided.

## SPLIT TOOTH

Complete, visible separation of the entire tooth into two parts, usually in a mesio‐distal direction.

### Prevalence

Split tooth (ST) is the result of the progression and expansion of a coronal crack resulting in mechanical failure of the crown and root, resulting in complete longitudinal separation of the tooth, usually in a mesio‐distal direction (American Association of Endodontists, 2022). The literature on the prevalence and presentation of ST is sparse (Schurz et al., 2020).

### Aetiology

ST is a progression of CT (Table 2).

### Clinical features

The tooth fragments are completely separated or separated with pressure. There may be symptoms of AP, including pain on biting, a narrow, isolated periodontal probing, or tooth mobility. Gentle probing of the ST occlusally will wedge the fragmented tooth apart (Table 3).

### Radiographic features

Visible separation into at least 2 fragments, the tooth is usually, but not exclusively, root filled. A periradicular radiolucency will be associated with the separated fragment.

### Management options, conclusion, and summary

ST has an unfavourable prognosis, and timely extraction should be considered to minimise the development of acute symptoms and limit bone loss.

## VERTICAL ROOT FRACTURES (VRFs) IN NON‐FILLED TEETH

An incomplete longitudinal (axial) root fracture, involving the cementum, dentine, and root canal space of non‐root‐filled teeth.

### Prevalence

The incidence of VRFs in non‐root‐filled teeth (NRFT) has been reported to be between 6% and 37%, with a predilection for males and patients over 50 years of age (Chan et al., 1998, 1999; Liao et al., 2017). The majority of research on VRFs in NRFT involves the Chinese population (Liao et al., 2021).

VRFs in NRFT mainly affect first molars, particularly the mesio‐buccal root of maxillary and the mesial root of mandibular molars (Liao et al., 2021). The fracture line typically originates from the root apex and appears on the buccolingual aspect of the root (Wang et al., 2012). Affected teeth are commonly (minimally) unrestored but have signs of excessive tooth surface loss, indicating repetitive and/or increased occlusal forces and/or an unfavourable cusp/fossae relationship (Chan et al., 1998; Yang et al., 1995).

### Aetiology

Multiple predisposing factors have been reported (Table 2). Certain dietary patterns, specifically relating to Asian populations, have been suggested to contribute to the higher incidence of spontaneous VRF in NRFT (Yang et al., 1995; Yeh, 1997).

### Clinical features

Teeth may be asymptomatic, have temperature sensitivity, have discomfort upon mastication, and/or have pulpitis or symptoms of AP (Bhaskar et al., 2011; Wang & Su, 2009; Yang et al., 2023).

An isolated, deep, narrow periodontal pocket is not as commonly identified in VRF of NRFT compared with VRF in root‐filled teeth (RFT) (Lee et al., 2023). Sensibility testing may reveal a positive or negative response, depending on the extent of the VRF and severity of the pulp necrosis (Chan et al., 1998; Liao et al., 2021; Yang et al., 1995).

### Radiographic features

A subtle or obvious fracture line within the root, periodontal ligament thickening, or periradicular bone loss adjacent to the separation of root fragments being evident radiographically (Chan et al., 1998; Yang et al., 1995). The European Society of Endodontology recommend small field of view CBCT may be used to confirm peri‐radicular signs of bone loss if clinical and/or PR assessment is inconclusive (Patel et al., 2019) (Table 3).

### Management options

Exploratory surgery may be indicated if the above findings are inconclusive and/or the nature and feasibility of management versus extraction need to be determined (Chan et al., 1998, 1999; Walton, 2017).

Early intervention is essential to limit the progression of VRF in NRFT and (further) bone destruction adjacent to the affected root. Extraction of single‐rooted teeth is recommended. Root resection (complete or partial) of the affected root may be an option in multirooted teeth depending on the position, level, and extent of the VRF. Endodontic treatment of the intact roots, followed by restoration of the access cavity with a direct plastic restoration, which should extend into the affected canal and apical to the planned root resection or amputation level. Cuspal coverage restoration is recommended (Ferracane et al., 2022; Kanamaru et al., 2017; Zhang et al., 2024). At present there is limited evidence to support intentional replantation and extraoral bonding for VRF.

### Conclusion and summary

The current evidence on treatment strategies for VRFs in NRFT is limited; more clinical studies with long‐term follow‐up are needed.

## VERTICAL ROOT FRACTURES (VRFs) IN ROOT FILLED TEETH

An incomplete longitudinal (axial) root fracture, involving the cementum, dentine, and root canal space of root‐filled teeth.

### Prevalence

VRF is more commonly associated with RFT than NFRT. The reported prevalence of extraction of RFT with VRF ranges from 4% to 32% (Lee et al., 2023; von Arx et al., 2021).

Maxillary (pre‐)molars and mandibular molars are the most commonly affected (Patel et al., 2022; PradeepKumar et al., 2016). The incidence of VRF increases with age, being more prevalent in 40+ year‐old patients (PradeepKumar et al., 2016; Yoshino et al., 2015).

### Aetiology

The aetiology is multifactorial; several putative predisposing and contributory factors have been reported (Table 2). The most critical factor(s) are unknown and difficult to elucidate. VRF is a common cause of extraction (13–21%) of RFT (Kim et al., 2024; Touré et al., 2011; Yoshino et al., 2015).

The pathogenesis of a VRF in RFT is complex and has not been clearly established. There are three main stages: crack initiation, propagation, and separation.

In the early stages, a crack may develop at either the coronal or apical aspect of the root and progress in either the apico‐coronal and/or bucco‐lingual aspects of the tooth. Unlike traumatic dental injuries, which are acute, high‐impact in nature, and usually present immediately after a recognised impact, VRFs develop as a result of a dynamic cyclical fatigue process over a long period of time (Kishen, 2015; Patel et al., 2022).

In advanced stages, continuous microbial ingress leads to biofilm formation and the loss of periodontal attachment adjacent to the fracture line (Walton et al., 1984). Complete longitudinal separation of the root eventually occurs, resulting in a split root (American Association of Endodontists, 2022).

### Clinical features

Presentation depends on the location, extent, and nature of the VRF. Early‐stage VRFs are a challenge to detect as the patient may be asymptomatic (Table 3).

There may be symptoms and/or signs of AP. Typical features of advanced VRF include an isolated, narrow, deep periodontal pocket (pathognomonic if detected on both sides of a root) and the presence of multiple sinus tracts (Patel et al., 2022) (Table 3).

Increased probing depths should be cautiously interpreted in the context of the history of the tooth and distinguished from probing attributed to periodontal disease and/or a sinus tract of endodontic origin (Lee et al., 2023).

Removal of existing restorations and root filling materials helps to determine the presence and/or extent of a VRF (Abbott, 2004). Removal of interproximal restorations allows probing of the mesial and distal surfaces, which may otherwise be inaccessible due to the presence of proximal contacts.

It is essential to use magnification (see CT section); if the VRF is still not readily visible, exploratory surgical procedures may be indicated. However, only fracture line(s) on the approximal root surfaces (i.e., buccal and lingual) will be accessible and visible.

### Radiographic presentation

The radiographic appearance (Table 3) of early‐stage VRFs may vary from no obvious bone loss to subtle periradicular bone loss, with long‐standing VRFs typically presenting with periradicular (J‐shaped) radiolucency and/or frank separation of the root (split root).

A CBCT may be indicated to confirm periradicular signs of bone loss if clinical and/or PR assessment is inconclusive (see VRF in NRFT section) (Chavda et al., 2014; Patel et al., 2019, 2022). CBCT cannot reliably detect VRFs within the root due to the image resolution being insufficient to detect fractures that are typically 50‐100 μm in width (Brady et al., 2014).

### Clinical management

The prognosis is poor (Fuss et al., 2001; Walton, 2017; Zadik et al., 2008). Prompt management is recommended as soon as diagnosis is reached to reduce the likelihood of acute AP symptoms and/or further periradicular bone breakdown, as this may complicate and/or delay dental implant treatment. Extraction is recommended in a single‐rooted tooth with a VRF (Patel et al., 2022).

In multi‐rooted RFT, root resection or root amputation may be considered as an alternative to extraction. A multi‐disciplinary approach is recommended in the treatment planning. There is no data on survival rates of root resection or root amputation for VRF in RFT; however, extrapolation from root resection on periodontally affected teeth has been reported to be 90.6% and 96.8% over 10 and 15 years, respectively (Derks et al., 2018; Fugazzotto, 2001).

### Preventive management

The prevention of VRFs is critical; identifying susceptible teeth and utilising conservative endodontic procedures together with expedient and appropriate post‐endodontic restorative procedures, is paramount to reducing the incidence of terminal VRFs (Patel et al., 2022). Minimising excessive removal of dentine during access cavity and canal instrumentation preserves pericervical dentine, which may reduce the impact of (para)functional occlusal forces (Clark & Khademi, 2010). Adhesive post‐endodontic restorative techniques are recommended to minimize dentine removal, and posts, if required, should be placed ‘passively’, and excessive ‘post‐space’ preparation should be avoided. After the root canal system has been obturated, the access cavity should be immediately restored with a resin‐based composite restoration. When indicated, prompt cuspal coverage restoration is essential to reduce the likelihood of VRF.

Proximal contacts help to dissipate occlusal forces. The replacement of missing posterior units is desirable to increase the number of occlusal and proximal contacts and distribute (non‐)occlusal loading (Gulabivala & Ng, 2023).

In cases of an RFT with a pre‐existing crack(s), the placement of bioactive or adhesive intracanal materials beyond the extension of crack(s) may reduce further propagation and/or microbial leakage (Davis & Shariff, 2019; de Toubes et al., 2024).

## UNIVERSAL CONSIDERATIONS FOR CT AND VRF

The impact of excessive occlusal forces cannot be overemphasised in the aetiology of CT, ST, and VRF. Consideration should be given to the opposing dentition, including their restoration status and occlusal topography. Fabrication of occlusal stabilisation splints and/or referral to relevant specialists should be considered for the management of parafunctional habits (Kui et al., 2020; Shehri et al., 2022).

Occlusal interferences on all, not just the affected teeth and/or restorations, should be managed appropriately. When indicated, composite resin canine ramps may help re‐establish canine guidance, allowing for posterior disocclusion in excursions, reducing lateral forces on these teeth, and lowering masticatory muscular activity (Abduo & Tennant, 2015). An interdisciplinary approach may be required for complex cases. It is essential to educate the patient of the prognosis and recommend reducing excessive occlusal loading on cracked RFT.

## CONCLUSION

Clinicians should be mindful of the significance, prevention strategies, and timely management of CT and VRF. Patients should be made aware of the part they play in limiting the CT and VRF. This is especially relevant as life expectancy as well as patients’ expectations are increasing. Periodic monitoring of (root filled) CT is essential for detecting early signs of periradicular bone loss, which may indicate an early stage VRF.

## AUTHOR CONTRIBUTIONS

Shanon Patel: conceptualisation, methodology, visualisation, resources, writing—original draft, writing—review and editing, project administration. Peng‐Hui Teng, Wan‐Chuen Liao, Matthew Craig Davis, Ales Fidler, Franziska Haupt, Ronald Ordinola Zapata, Rahul Bose. Cristiano Fabiani, writing—review and editing.

## CONFLICT OF INTEREST STATEMENT

The authors declare no conflicts of interest.

## ETHICS STATEMENT

None.

## Data Availability

Data sharing is not applicable to this article as no new data were created or analyzed in this study.
